# The Implicitome: A Resource for Rationalizing Gene-Disease Associations

**DOI:** 10.1371/journal.pone.0149621

**Published:** 2016-02-26

**Authors:** Kristina M. Hettne, Mark Thompson, Herman H. H. B. M. van Haagen, Eelke van der Horst, Rajaram Kaliyaperumal, Eleni Mina, Zuotian Tatum, Jeroen F. J. Laros, Erik M. van Mulligen, Martijn Schuemie, Emmelien Aten, Tong Shu Li, Richard Bruskiewich, Benjamin M. Good, Andrew I. Su, Jan A. Kors, Johan den Dunnen, Gert-Jan B. van Ommen, Marco Roos, Peter A.C. ‘t Hoen, Barend Mons, Erik A. Schultes

**Affiliations:** 1 Department of Human Genetics, Leiden University Medical Center, Leiden, The Netherlands; 2 Department of Medical Informatics, Erasmus University Medical Center Rotterdam, Rotterdam, The Netherlands; 3 Dutch Techcentre for Life Sciences, Utrecht, The Netherlands; 4 Leiden Institute for Advanced Computer Science, Leiden, The Netherlands; 5 Department of Molecular and Experimental Medicine, The Scripps Research Institute, La Jolla, CA, United States of America; 6 STAR Informatics / Delphinai Corporation, Port Moody, BC, Canada; Garvan Institute of Medical Research, AUSTRALIA

## Abstract

High-throughput experimental methods such as medical sequencing and genome-wide association studies (GWAS) identify increasingly large numbers of potential relations between genetic variants and diseases. Both biological complexity (millions of potential gene-disease associations) and the accelerating rate of data production necessitate computational approaches to prioritize and rationalize potential gene-disease relations. Here, we use concept profile technology to expose from the biomedical literature both explicitly stated gene-disease relations (the explicitome) and a much larger set of implied gene-disease associations (the implicitome). Implicit relations are largely unknown to, or are even unintended by the original authors, but they vastly extend the reach of existing biomedical knowledge for identification and interpretation of gene-disease associations. The implicitome can be used in conjunction with experimental data resources to rationalize both known and novel associations. We demonstrate the usefulness of the implicitome by rationalizing known and novel gene-disease associations, including those from GWAS. To facilitate the re-use of implicit gene-disease associations, we publish our data in compliance with FAIR Data Publishing recommendations [https://www.force11.org/group/fairgroup] using nanopublications. An online tool (http://knowledge.bio) is available to explore established and potential gene-disease associations in the context of other biomedical relations.

## Introduction

Organizing the knowledge space around disease pathophysiology and phenotypes is crucial for data interpretation [1,2] and translational medicine [3]. Presently however, most geneticists attempt to rationalize large and complex datasets using keyword-based searches to interrogate existing knowledge resources. Although keywords can effectively retrieve individual documents, they fail to scale with either data complexity or data volume. For keyword searches to work comprehensively, ambiguities in terminology and meaningful relations between keywords must be factored in by the human operator. Without constructing sophisticated queries, large troves of relevant information typically remain elusive to the clinician and researcher.

In contrast to keyword approaches, we use concept profiles to expose the associative information contained in MEDLINE [http://www.ncbi.nlm.nih.gov/pubmed] abstracts as a document-independent, weighted semantic network of disambiguated biomedical concepts. From this network we then expose all gene-disease associations forming a “Literature Wide Association Study” or LWAS. The vast majority of gene-disease associations are implicit, that is, they are associated by their mutual association to intermediate concepts. We call the network of implicit relations the gene-disease implicitome.

Concept profile technology behind LWAS has its roots in Swanson’s hidden relationship model [4], where it is assumed that one set of literature contains direct relationships between concept X and concept Y, while a separate disjoint set of literature contains direct relationships between concept Y and concept Z. Combining these literature sets permits the inference of a relationship between X and Z via the linking concept Y [5][6]. This approach has been previously applied in the functional annotation of proteins [7], interpretation of gene expression data [8–11], chemical toxicological classification [12], and protein-protein interaction prediction [13]. In the present study, we exploit the linking Y concepts to inform and rationalize the putative gene-disease relations. More specifically, in our case, the gene would be the X concept and the disease the Z concept. The Y concepts can belong to any biomedical concept category, such as another gene or disease, or a biological process, phenotype, or drug.

Here, we use LWAS to show how information in the concept profiles can be used to prioritize and interpret new gene candidates implicated in disease. For example, this information can be used to build cases for causality in pathogenicity or cases for genetic modifiers. We first validate our LWAS by manually analyzing 105 top-ranking potentially novel gene-disease associations for relevant information that implicates genes in the etiology of disease phenotypes. We also report on a detailed case study involving causative genes for the rare genetic disorder Seckel Syndrome. Then in a retrospective analysis, we show how to appropriately integrate LWAS with Genome Wide Association Study (GWAS) data (14) so that empirically derived associations can be rationalized using the totality of the biomedical literature. Together, these analyses demonstrate that LWAS complements other data sources when interpreting the biomedical significance of newly reported gene candidates causing disease or contributing to pathophysiology.

## Results

### LWAS: Concept profile analysis exposes implicit gene-disease associations

**Fig 1:**
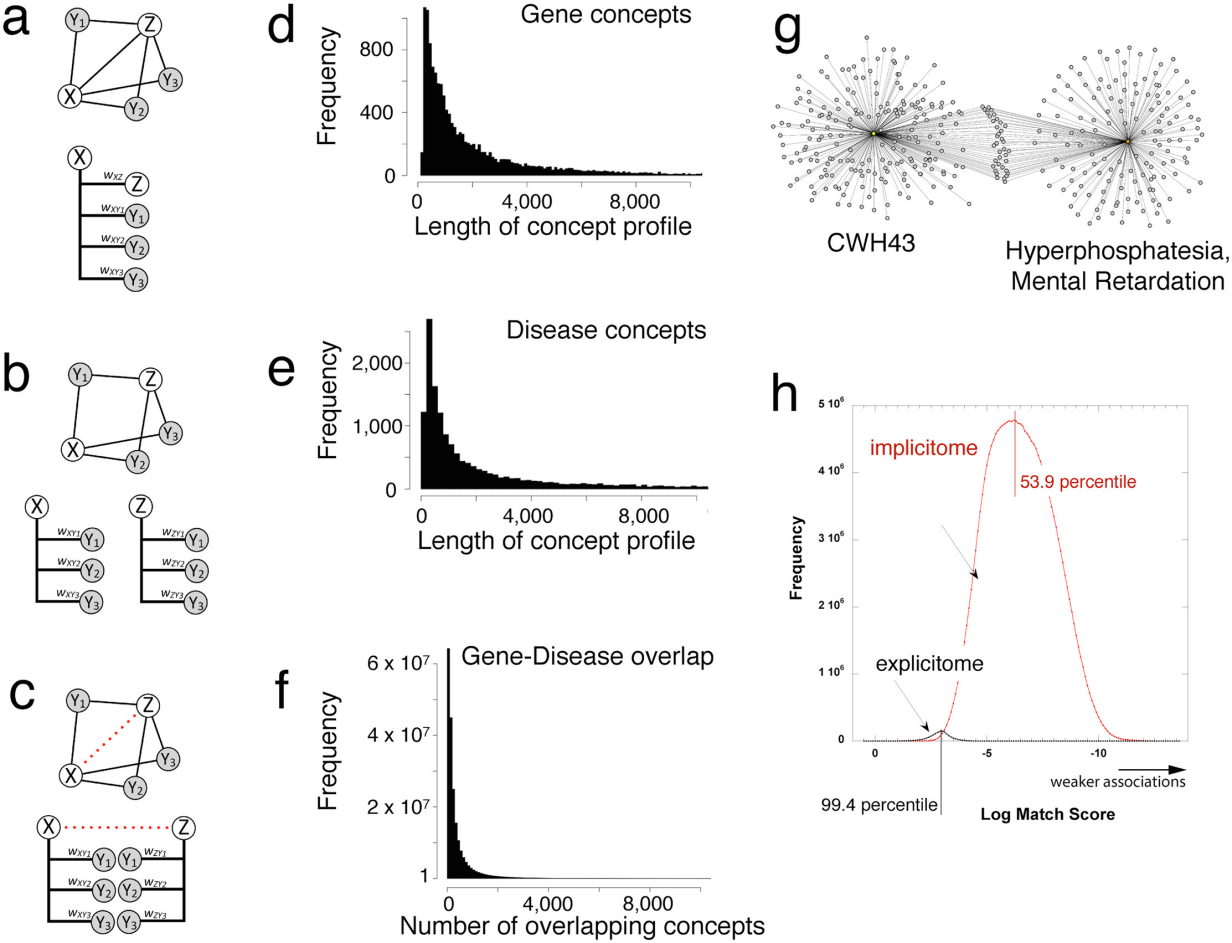
Gene-Disease LWAS using concept profiles and networks of implicit information. **a)** Concepts X and Z share an association in a hypothetical concept network via an explicit link (co-occurrence) and multiple implicit links (indirect connections via an intermediate concept, Y1, Y2, and Y3). The concept profile for concept X is depicted where the weights (w) between concepts reflect the co-occurrence frequencies of each concept in the data source. **b)** Concept profiles for concepts X and Z have explicit links to concepts Y1, Y2, and Y3 but no explicit link between themselves, as reflected in their corresponding concept profiles. **c)** The intermediate shared concepts between concept profiles X and Z constitute implicit information, indirectly linking X and Z (red dotted line). The strength of the implicit link (match score) is computed as the inner product of the weights of matching concepts in the concept profiles. **d & e)** The distribution of concept profile size for gene (median 1142, maximum 56,028) and disease (median 995, maximum 81,562) concepts. **f)** The distribution of number of overlapping concepts between gene and disease concept profiles (median 180, maximum overlap 40,725). Only 23 concept pairs had no overlapping concepts. **g)** Concept profiles for the human gene *CWH43* (left) and the disease “Hyperphosphatesia with Mental Retardation” (right) which share no explicit co-occurrence. The 37 overlapping concepts are shown clustered in between. Both the number and weights of these overlapping links contribute to the strength of the implicit association. **h)** The distribution of match scores (higher numbers indicating stronger associations) for the 204 million LWAS-derived gene-disease pairs for both the explicit (black) and implicit (red) associations.

A detailed description of concept profile technology can be found in Materials and Methods. Briefly, there are three steps to the analysis: (1) We index the biomedical terminology in a large corpus of MEDLINE abstracts (dating from January 1980 to July 2014) using an extensive thesaurus, mapping and disambiguating terms to over 600,000 biomedical concepts; (2) For each recognized gene and disease concept in MEDLINE a weighted list (profile) of all other concepts is constructed from the observed co-occurrence frequency in each abstract (Fig 1); (3) The strength of the association between a pair of concepts, i.e., the match score, is determined by both the number and the weights of the overlapping concepts between their profiles.

Of the 417,561,711 possible gene-disease pairs (19,113 genes x 21,847 diseases in our thesaurus) more than half (213,489,335) lacked sufficient literature representation to build a concept profile for either one or both of the concepts in the pair (we address the heterogeneity in literature abundance below). Match scores were computed for the remaining 204,072,376 pairs and normalized to a percentile. Typically, gene-disease pairs exposed by concept profiles share a few hundred overlapping concepts, but in some cases we found overlaps that extended to tens of thousands (Fig 1f and 1g). Importantly, concept profiles indicate the individual contribution of each concept to the overall match score. In the detailed examples of gene-disease associations below, we show how this information can be very useful when rationalizing the gene-disease associations.

Explicit associations (a gene and disease that co-occur in the same abstract) account for only 0.73% (1,479,895) of the total number of associations. The match scores of the explicit associations are among the strongest, with the distribution peaking in the 99.4 percentile (Fig 1h, black line). The vast majority of the associations, however, are implicit (Fig 1h, red line). Implicit gene-disease associations generally have lower match scores than explicit associations, but there is nonetheless substantial overlap between the two distributions. Hence, there are numerous gene-disease associations that have not been made explicit in the literature but for which there is strong evidence of a meaningful relation. In the range of the nearly 1.5 million explicit gene-disease match scores (extending to the 86th percentile), there are more than 25 million implicit associations (S1 Table).

**Fig 2:**
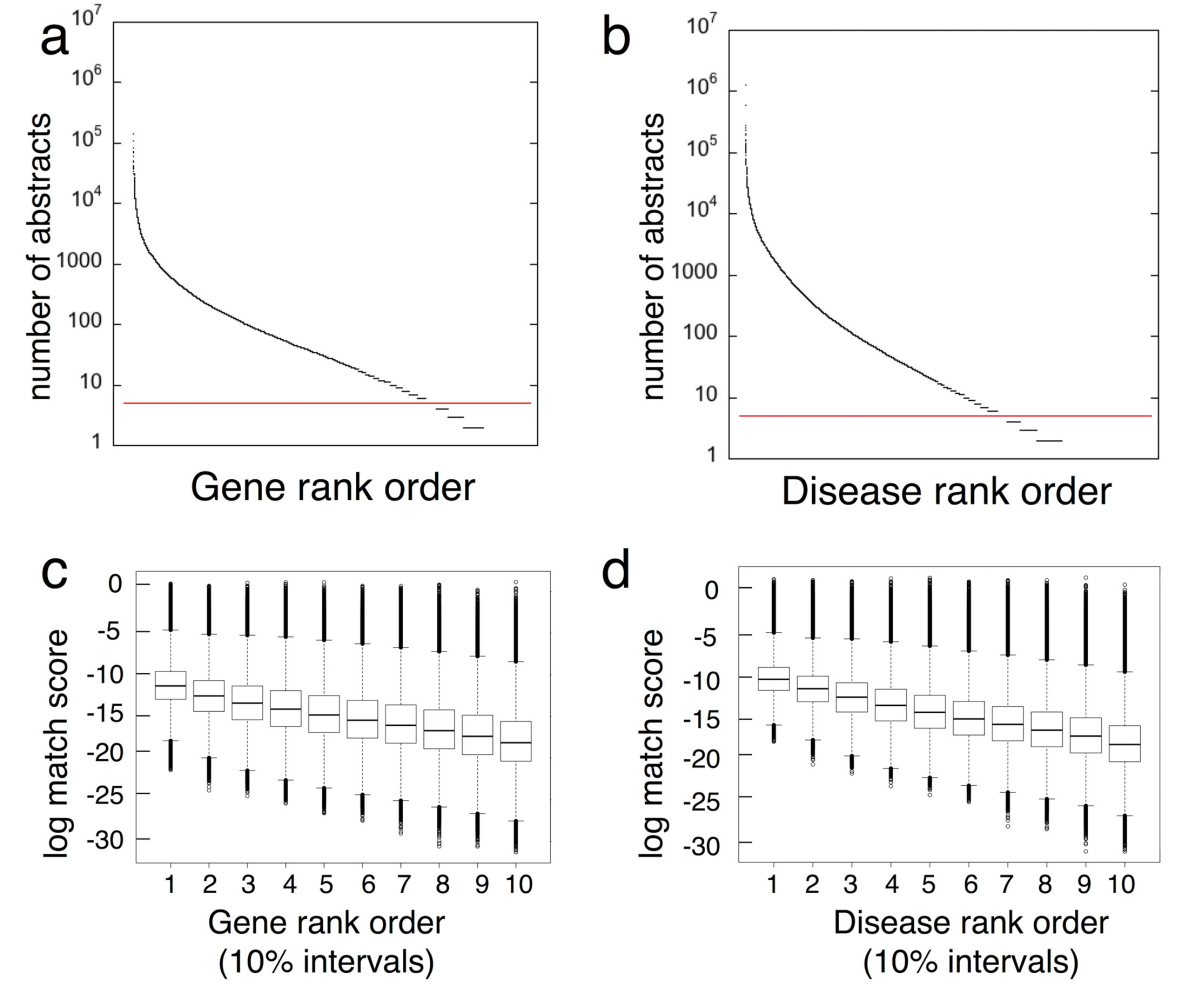
Correction of literature bias in the match score. **a,b**) Distribution of genes and diseases recognized by LWAS when sorted by publication abundance (log number of MEDLINE abstracts). Red lines indicate the 5-abstract cut-off, below which concept profiles are not constructed. **c,d)** Distribution of gene and disease rank orders, binned in 10 percentile intervals (x-axis). Higher numbers indicating stronger associations (y-axis).

Some genes and diseases are more extensively studied than others and this is reflected in the biomedical literature (S2 and S3 Tables). Fig 2a and 2b depict the MEDLINE abundance of gene and of disease concepts as rank-ordered distributions of the number of abstracts in which they are mentioned. The biomedical literature is biased toward publications mentioning a relatively small number of commonly studied genes and diseases (steep part of curve on the left). A relatively small number of genes (fewer than 10%) and diseases (fewer than 5%) account for 80% of all MEDLINE publications. Although match score tends to decrease with publication abundance there are millions of gene-disease pairs having low publication abundance yet match scores in the same range as gene-disease pairs that are highly published (Fig 2c and 2d). Hence, concept profiles can in many cases compensate for strong biases in literature abundance.

We have made individual gene-disease associations and their match scores accessible as a public data set and via an online tool (see Materials and Methods).

### Validation of the top LWAS associations

**Fig 3:**
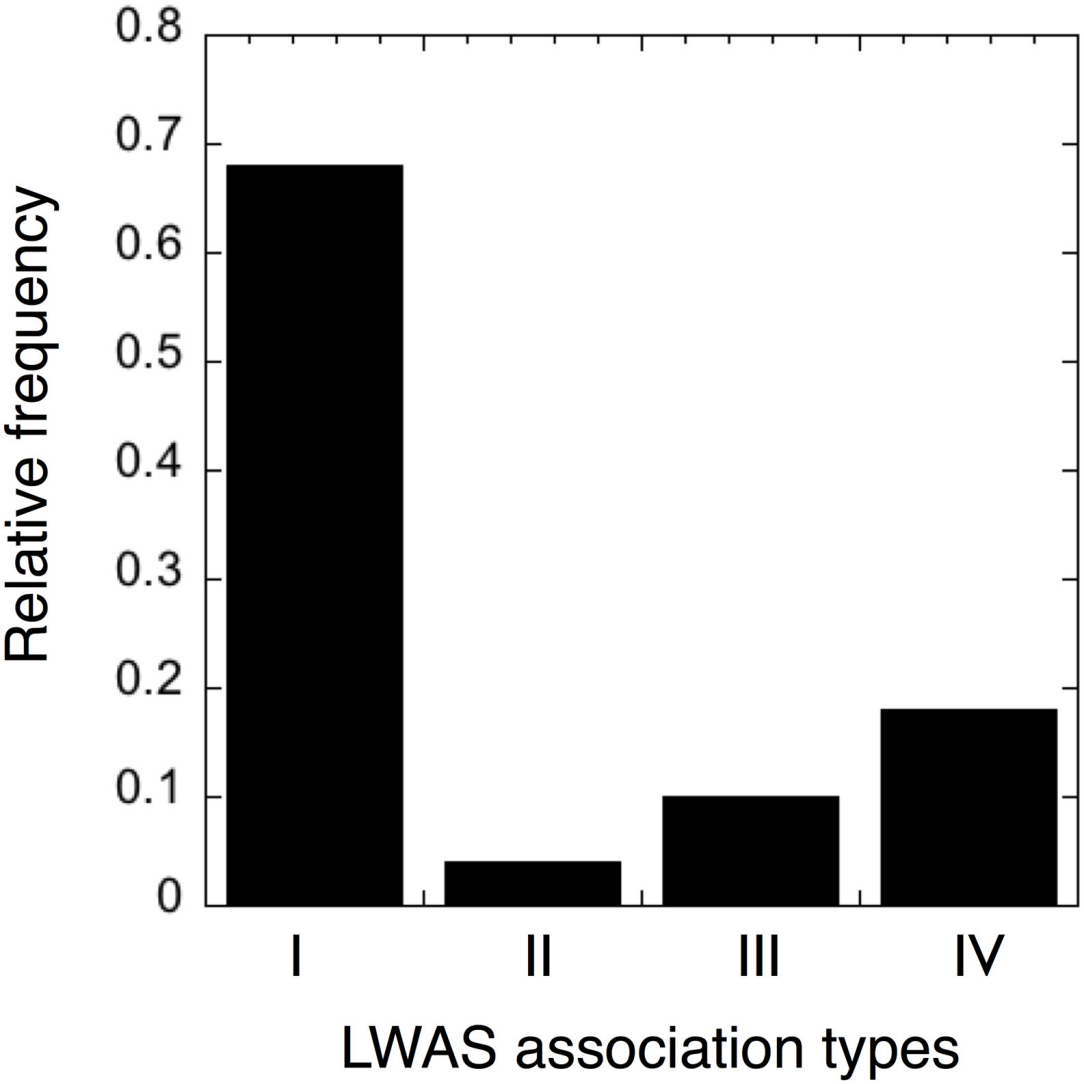
The relative distribution of LWAS association types. Distribution of the top 105 highest-ranking implicit gene-disease pairs determined by manual inspection: *Type I Gene family member* (n = 71) represents gene-disease associations where a family member of the gene is causing the disease or a disease with very large phenotypic overlap; *Type II Negation* (n = 4) and *Type III Homonym* (n = 11) represent different classes of LWAS false positives composing 14% of the cases. *Type IV Novel association* (n = 19) indicates gene-disease associations of promise for follow up investigations.

The 105 highest ranking implicit gene-disease associations and their top connecting concepts are listed in S4 Table. By applying our manual assessment procedure described in the Materials and Methods section, we found that the associations could be grouped into four types (Fig 3). Type I: gene-disease associations where a family member of the gene is causing the disease (71 cases; for example the gene-disease pair *TTBK1*-SCA11, where *TTBK1* and *TTBK2* are isoforms of the *TTBK* gene, and a mutation in the *TTBK2* form is causing the disease [14] or where a disease has a very broad phenotypic overlap with other disorders, e.g. the gene-disease pair *WDR62*-MCPH4, where MCPH4 is a subtype of autosomal recessive primary microcephaly (MCPH) for which *WDR62* is already found to be the causing gene for subtype MCPH2 [15], and the second most mutated gene in a large family study of MCPH [16]). These types of candidates are not necessarily false positives but can frequently be labeled as obvious. Type II: false positive gene-disease associations resulting from co-occurrences reflecting negative findings reported in the literature (4 cases). Type III: false positive gene-disease associations resulting from concept mapping errors, such as homonyms (11 cases). Type IV: Gene-disease associations that are novel and show promise for follow up investigations (19 cases).

Although the false positive results among the high scoring gene-disease associations (14%) confirm that improvements can still be made in the automatic mapping of biomedical concepts [17], Type IV results demonstrate that LWAS is nevertheless exposing potentially millions of biomedically important yet still unknown gene-disease associations and the relevant information for rationalizing them. To illustrate, we mention here two examples of Type IV associations: First, the gene *CYP2R1* had a strong association with Smith-Lemli-Opitz (SLO) syndrome. The top-ranking connecting concepts are 7-dihydrocholesterol reductase (*DHCR7*; defects in this gene cause SLO syndrome [18,19] and cholesta-5,7-dien-3beta-ol (= 7-dihydrocholesterol). A pathological hallmark of SLO is increased levels of 7-dihydrocholesterol [20]. 7-dihydrocholesterol is a precursor of vitamin D3 and defects in *CYP2R1* are known to affect vitamin D3 levels [21]. Thus, LWAS implicates *CYP2R1* in SLO syndrome since defects may potentially lead to 7-dihydrocholesterol accumulation.

In the second Type IV example, *CWH43* is linked to the disorder hyperphosphatasia with mental retardation. The top ranking connecting concept is *PGAP2*. *PGAP2* defects cause Hyperphosphatasia with mental retardation syndrome 3 and are thought to be related to the function of *PGAP2* in the maturation of glycosylphosphatidylinositol (GPI) modifications to proteins that anchor them in the plasma membrane [22,23]. *CWH43* is a gene that has not yet been connected to disease but its protein product has sequence homology with *PGAP2* and has been demonstrated to be involved in remodeling of the lipid moiety of the GPI anchor in yeast [24].

### LWAS assisted rationalization of causative genes for Seckel Syndrome

LWAS can expose information that implicates genes in the etiology of disease phenotypes. Focusing on the genetic disorder Seckel Syndrome [MIM 210600], we perform a 5-year retrospective analysis using a Medline corpus of abstracts published before July 2009. By looking backwards in time, we demonstrate how overlapping concepts between gene and disease concept profiles can help expert researchers to identify and functionally rationalize the role of candidate genes in disease pathophysiology.

Seckel Syndrome is a rare autosomal recessive disorder characterized by growth retardation, mental retardation, microcephaly, and a characteristic “bird-headed” facial appearance (receding forehead and micrognathia with a prominent nose). Seckel syndrome shows phenotypic overlap with type II microcephalic osteodysplastic primordial dwarfism (*MOPD2*, MIM:210720).

In 2009, the OMIM database indicated the following loci for Seckel Syndrome: chromosomes 18p11-q11 (*SCKL2*, MIM:606744), 14q (*SCKL3*, MIM:608664) and 13q12 (*SCKL4*, MIM:613676), but causative mutations were only found in a single gene: *ATR* (ataxia-telangiectasia and RAD3-related protein, *SCKL1*) [25]. As expected, LWAS identified *ATR* as the highest-ranking gene associated with Seckel Syndrome, mainly based on explicit links (direct co-occurrences in seven Medline abstracts, Table 1). Table 1 ranks genes having high match scores to Seckel Syndrome based on overlapping concepts in the concept profiles generated until July 2009. Implicit links (no co-occurrences) are in bold font.

**Table 1:** Ranked list of genes having high match scores to Seckel Syndrome based on overlapping concepts in their concept profiles generated until July 2009. *NUP85* is ambiguous, with PCNT as the synonym causing a homonym problem with the *PCNT* gene, and a large overlap of articles. ANTXR1 was previously labeled as ATR, causing the same sort of problem as for NUP85, with an overlap of articles with ATR. Only *ATR* had been identified as a causative gene for Seckel Syndrome by July 2009. Bold formatting indicates gene-disease associations derived by implicit information only (i.e., having no co-occurrences in the literature up to July 2009).

Rank	Gene name	Co-occurrences in MEDLINE abstracts until July 2009	Top-ranking concept explaining link between gene and Seckel syndrome	Causative gene for Seckel syndrome	PMIDs and publication date of publication (italics) describing causative mutations in that gene
1	*ATR*	7	*ATR*	Yes	19504344,16015581,15616588,15496423,15309689,14571270,*12640452*; April 2003
2/3	*PCNT*	3	Seckel syndrome		19546241,18157127,16278902
2/3	*NUP85*	2	Seckel syndrome		19546241,18157127
4	*ANTXR1*	2	*ANTXR1*		19504344,12640452
5	*MCPH1*	3	*MCPH1*		19546241,17102619,16217032
6	*NBN*	2	*NBN*		18664457,15616588
**7**	***MCPH2***	**0**	**Primary microcephaly**		*Implicit link*: *no co-occurrence in Medline up to July 2009*
8	*FANCD2*	2	*FANCD2*		15616588,15314022
**9**	***ATRIP***	**0**	***ATR***		*Implicit link*: *no co-occurrence in Medline up to July 2009*
10	*DNMT1*	1	*DNMT1*		17015478
11	*MDC1*	1	*MDC1*		18664457
12	*FANCC*	1	Fanconi's Anemia		10232749
13	*PALB2*	6	Fanconi's Anemia		17224058,15314022,10232749,7686032,6465473,3115102
**14**	***CENPJ***	**0**	**Microcephaly**	**Yes**	*Implicit link*: *no co-occurrence in Medline up to July 2009*. ***20522431*; June 2010**
**15**	***ASPM***	**0**	**Microcephaly**		*Implicit link*: *no co-occurrence in Medline up to July 2009*
16	*CHEK1*	5	*CHEK1*		19504344,18077418,17015478,16217032,15616588
**17**	***PROP1***	**0**	**dwarfism**		*Implicit link*: *no co-occurrence in Medline up to July 2009*
18	*MMAB*	2	*MMAB*		19504344,15314022
**19**	***TOPBP1***	**0**	***ATR***		*Implicit link*: *no co-occurrence in Medline up to July 2009*
20	*FOXL2*	1	*FOXL2*		16015581

The second highest-ranking LWAS gene after *ATR* in Table 1 is pericentrin (*PCNT*). In 2009 there were three articles where *PCNT* and Seckel syndrome were mentioned in the same abstract. One of them actually claims that mutations in *PCNT* can cause Seckel Syndrome [26]. It is now believed that mutations in *PCNT* cause the phenotypically very similar disease Primary microcephaly and microcephalic osteodysplastic primordial dwarfism type II (MOPD II). LWAS postulates many other genes associated with primary microcephaly, such as *MCPH1* (rank 4), *MCPH2* (rank 7), *ASPM* (rank 15), and must therefore also be considered as candidates causing or impacting the development of Seckel syndrome. This is to be expected since microcephaly is an important pathological hallmark of Seckel syndrome. Additional genes associated with Seckel syndrome were suggested by LWAS through function in the cell cycle check point. This is also in line with the current knowledge about the molecular pathophysiology of Seckel syndrome, as all causative genes known so far indeed have a function in the control mechanisms that ensure the fidelity of cell division.

Additional gene mutations causing Seckel syndrome were published in the period after July 2009. These gene mutations were highly ranked in the LWAS using literature before that date (Table 1). For example, in June 2010 *CENPJ* was published as a causative gene for Seckel syndrome. Concept profiles showed *CENPJ* ranked 14th out of 12,391 genes having concept profiles, and as the third gene without direct co-occurrences (Table 1). The concept microcephaly contributed the most to the *CENPJ*-Seckel Syndrome match score (Table 2). Microcephaly is an important pathological feature of Seckel syndrome and *CENPJ* already had been associated with microcephaly in April 2005 [27]. In the terms of Swanson’s relationships model, Microcephaly would be the key linking Y-concept.

**Table 2:** Top ranking overlapping concepts between Seckel Syndrome & *CENPJ*. The contribution of each concept to the overall match score is given as a percentage.

Rank	Overlapping concept	Identifier	Contribution (%)
1	Microcephaly	UMLS:C0025958	17.77
2	Primary microcephaly	UMLS:0431350	17.31
3	*MCPH1*	OMIM:251200	11.86
4	*Mcph1*	EG:244329	11.44
5	*MCPH1*	EG:36272	11.44
6	*MCPH1*	EG:100125976	11.41
7	*MCPH1*	EG:79648	7.54
8	*PCNT*	EG:5116	4.38
9	osteodysplastic primordial dwarfism	UMLS:C0432244	1.94
10	*NUP85*	EG:79902	1.11
11	MOPD II	OMIM:210720	0.93
12	pericentrin	UMLS:C0252534	0.77
13	Dwarfism	UMLS:C0013336	0.72
14	Centrosome	GO:0005813	0.55
15	Genes, Recessive	UMLS:C0017361	0.32

More recently (January 2011-February 2014), mutations in for example *RBBP8*, *CEP152* and *DNA2* were found to cause Seckel syndrome: *SCKL2*, October 2011 [28]; *SCKL5*, January 2011 [29]; *SCKL8*, February 2014 [30], respectively. In 2009, *RBBP8* ranked 209/12,391. The highest ranking concepts for the implicit association of *RBBP8* and Seckel Syndrome were *ATR* and cell cycle checkpoint. *DNA2* ranked 296/12,391 with *ATR*, *MEC1*, and replication fork as linking concepts, implicating genes with a function in cell cycle control (similar to *ATR*). Unfortunately, in 2009 we could not have detected the relation between *CEP152* and Seckel Syndrome, since we had no concept profile for *CEP152* due to its low literature abundance at the time (having fewer than 5 abstracts). *CEP152* is thus an example of how an overall scarcity in the existing genetic knowledge space effectively delimits a deductive approach in identifying causative genes for Seckel Syndrome.

The contribution of an *ATRIP* variant to Seckel syndrome has been reported, but not confirmed [31]. *ATRIP* was the second highest ranking gene in 2009 with no co-occurrence with Seckel syndrome (Table 1). *ATR* was the top connecting concept, which is logical since the gene product of *ATRIP* is the *ATR*-interacting protein *ATRIP*.

### LWAS-assisted interpretation of GWAS hits

To illustrate how implicit information can be used to complement gene-disease associations obtained using other independent approaches, we analyzed the overlap between LWAS and GWAS. To do this, we focused on GWAS hits from the NHGRI GWAS Catalog spanning the interval between January 2013 and August 2014 (according to the PubMed publication date [http://www.ncbi.nlm.nih.gov/pubmed]), and compared this to LWAS from a literature corpus spanning 1980 until July 2012 to ensure that our literature associations were not influenced by the reporting of the GWAS hits in the literature. We applied multiple filters to the GWAS Catalog in order to achieve a comparable set of GWAS-LWAS gene-disease associations. A detailed description of these filtering steps is given in the Materials and Methods section.

**Fig 4:**
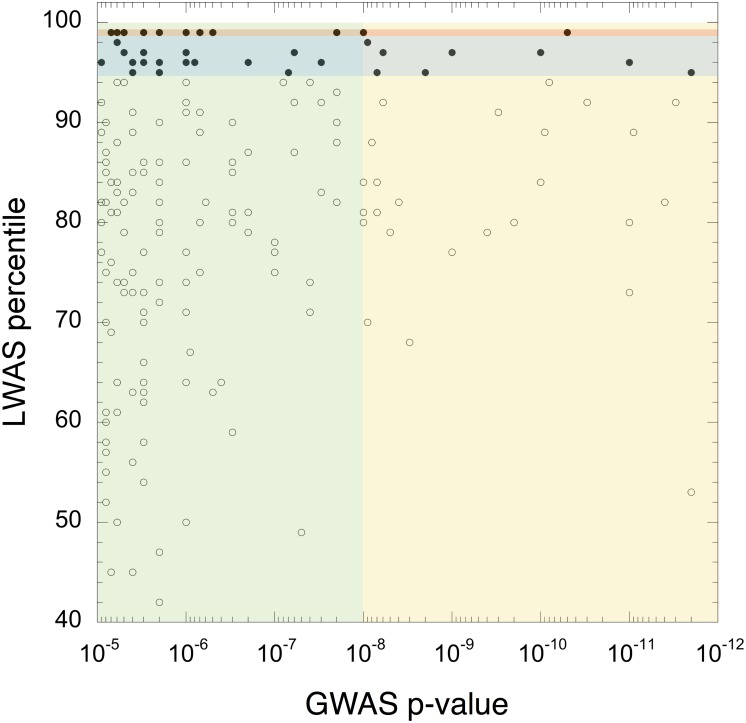
Overlapping implicit gene-disease associations between LWAS and GWAS. Green area: GWAS p-value cutoff of 10^−5^, yellow area: GWAS p-value cutoffs of 10^−8^, red horizontal area: LWAS 99^th^-percentile cutoff, blue horizontal area: LWAS 95^th^-percentile cutoff.

The GWAS Catalog filtering steps resulted in 238 GWAS records representing 35 diseases, all published in 2013 or later (S5 Table). Of these gene-disease associations, 194 had concept profiles and could be exposed from LWAS. 45 of these 194 gene-disease pairs (23%) pass the 95-percentile cutoff (4.6-fold enrichment; 5% would be random). At the 99th percentile, there were 12 (6%) gene-disease pairs (6-fold enrichment; 1% would be random). The overlap between these independent datasets can be quantitatively defined by partitioning the data based on the stringency of the associations. For GWAS, this includes the commonly used p-value cutoffs of 10^−5^ and 10^−8^. For LWAS this includes the 99^th^-percentile cutoff and 95^th^-percentile cutoff. Fig 4 depicts the correspondence of LWAS and GWAS rankings, for the overlapping gene-disease associations. These LWAS hits can be used to automatically focus relevant knowledge in the scientific literature to assist the expert in the interpretation of novel GWAS associations. For example, the gene-disease association with the most stringent p-value and highest percentile score from our study is *ERAP1* and Behçet’s disease. The overlapping concepts with the highest contribution score (HLA-B, 53% and Ankylosing spondylitis, 25%) are explicitly mentioned as evidence in the eventual landmark GWAS paper published after 2012 [32]: HLA-B*51 (a HLA-B serotype) as interacting with *ERAP1*, and *ERAP1* as one of the three risk loci shared with Ankylosing spondylitis and psoriasis, which are thought to involve pathogenic pathways similar to Behçet’s disease.

Importantly, of the 45 overlapping LWAS-GWAS hits above the 95th percentile, there were 16 gene concepts appearing in fewer than 100 abstracts. This result demonstrates the power of LWAS in providing relevant information even for rarely published genes (S5 Table).

## Discussion

We have shown that LWAS, a method that is based solely on the information available in the literature, can be used in conjunction with experimental data resources to interpret known associations, to anticipate novel associations, and to provide context and rationalization for both known and novel associations. The relative proportion of LWAS gene-disease associations that are novel and show promise for follow-up investigations is likely to decrease with lower match scores. However, as the vast majority of gene-disease associations have yet to be explicitly stated, the implicitome provides the broadest possible knowledge base in which gene-disease pairs can be interpreted. Investigating how properties such as network statistics and graph visualization techniques can aid in the automatic reproduction of the different types of LWAS categories currently determined by hand (type I-IV, described in the results section) is a topic for future work.

GWAS hits are the outcome of high-throughput screens having no dependence on prior biomedical knowledge. Thus, the correspondence of implicit LWAS associations to subsequently discovered GWAS associations indicates that the corpus of biomedical knowledge is tracking, and in some cases may even be anticipating future biomedical discovery. LWAS, therefore, complements GWAS: LWAS can detect associations for GWAS hits that are not yet sufficiently powered, and can provide information helping to rationalize mechanisms of pathogenicity; GWAS, on the other hand, has the potential of finding completely novel associations for which there are few or no antecedents in the literature. When integrated, LWAS and GWAS become a powerful tool allowing experts to intelligently prioritize gene-disease pairs for further study. Thus, tapping the implicitome to provide context and supporting evidence is of vital importance when new gene-disease associations are uncovered by high-throughput approaches.

Our application of concept profiles has clear limitations. Firstly, the underlying thesaurus used for mapping the gene and disease concepts in text is based on numerous integrated dictionaries. Keeping such a dictionary up to date with a minimal amount of identifier mapping errors is cumbersome and time-consuming. Therefore we are looking into other, open resources as basis for thesaurus creation and maintenance such as the BioPortal repository of biomedical ontologies [33] and WikiData [http://www.wikidata.org], which are offering other integrated knowledge sources in addition to the Unified Medical Language System (UMLS).

Secondly, while the bioinformatics community has made great progress in the normalization of gene mentions (mainly thanks to the BioCreative challenges [http://www.biocreative.org/]), disease mentions remain notoriously difficult to normalize [34]. Although our concept recognizer Peregrine has been shown to outperform MetaMap [35], a standard tool developed by the NLM for mapping UMLS metathesaurus concepts from text [36] our list of gene-disease associations nonetheless contains both false positives arising from errors in concept mapping as well as false negatives arising from errors due to incomplete thesaurus coverage and concept mapping. In our analysis of the top 105 gene-disease associations, we found 11 errors related to concept mapping. This number is expected to vary depending on the disease and gene, since some disease and gene names are more ambiguous than others. Recent research suggests that generation of word-concept statistical models from a knowledge base can aid in the task of word sense disambiguation [37], but the scaling of these models is not well studied and integration of these types of models into concept mapping systems awaits future investigation. As a way to indicate concept ambiguity in our thesaurus, we currently report on the number of within-thesaurus case-insensitive homonyms for each concept (see the field “homonymFrequency” in for example http://rdf.biosemantics.org/emco/v1.5/concepts/C1836621). More advanced methods can be envisioned [38] but in any case LWAS is vastly more efficient than digging up that same information by manual keyword searches of the literature.

Thirdly, the information in the literature is biased towards a relatively small number of commonly studied genes and diseases. Consequently, more than half of the possible gene-disease pairs from our thesaurus lacked sufficient literature representation to build a concept profile for either one or both of the concepts in the pair. Still, our resource is more than 400 times larger than a very recent gene-disease association database generated by the kernel-based machine learning method BeFree [39]. This difference might partly be explained by the sentence-based scope of the previously mentioned method, while our method relies on co-occurrence statistics on the abstract level. Furthermore, in LWAS, the focus lies not on prediction but rather on exposing all the information knowledge space as it currently exists. Integrating results from LWAS with machine learning approaches [39] and semantic interpreters [40] is a topic for future research. As a first step in this direction our Knowledge.Bio application [http://www.knowledge.bio/] integrates the top one percent of our gene-disease associations from the Implicitome with SemMedDB [41]. Starting with a gene-disease association hypothesis, Knowledge.Bio can be used to rationalize the association as being causative, or part of the pathogenic pathway that can possibly be influenced by drugs. Instead of reading all the literature on SLOS (788 citations in PubMed as of November 6, 2015) and CYP2R1 (190 citations in PubMed as of November 6, 2015) separately, the Y concepts gives a starting point for exploration using directed statements from SemMedDB. The filtering options in knowledge.bio allows for steering the exploration towards for example either more evidence for casuality or possible treatment options (for an example on SLOS, see [http://www.slideshare.net/goodb/poster-knowledgebio-an-interactive-tool-for-literaturebased-discovery]).

Biases in literature coverage reflect powerful historical trends in research and funding priorities in the life sciences. Yet, the uncertainty coefficient used to compute weights in concept profiles, as well as the large numbers of implicit links composing the semantic network (number of overlapping concepts between concept profiles) mitigate the effects of literature abundance biases with respect to the information content carried by the network. Hence, even rarely studied gene or disease concepts can have high match scores if they have enough overlapping concepts, as demonstrated in the GWAS study. Adding information from the numerous structured databases now available might fill some of the knowledge gaps surrounding rarely studied diseases and/or genes. Towards this end, we have adopted the nanopublication model [42,43] to facilitate the dissemination and integration of LWAS data with other data resources. A nanopublication links a description of the gene-disease association, its percentile-rank in this LWAS, and provenance metadata as a stand-alone semantically enabled publication. LWAS nanopublications can be queried and cited, and are machine-readable, thus interoperating with other semantic data resources.

Although we publish here LWAS-derived gene-disease associations as nanopublications, it is possible to expose from MEDLINE associations for any other concept pair (for example protein-protein [13], gene-chemical [12], or drug-drug [44]). Hence, LWAS has fundamental and universal application in any biomedical data-integration effort. S6 Table lists examples of resources directly compatible with the nanopublication format.

Prior knowledge, whether it is abundant or sparse (and even in cases where gene or disease concept profiles could not be constructed) is always an important factor when triaging millions of theoretically possible gene-disease associations arising in high-throughput empirical studies., By defining the boundaries of knowledge space (some 400 million possible gene-disease associations) and mapping those subject areas that are insufficiently studied, LWAS can provide research priority recommendations that could optimize discovery returns on investment [45].

## Conclusions

We have demonstrated the application of LWAS in rationalizing the implicit links between genes and diseases. The implicitome provides a rich theoretical, practical and historical context for interpreting data from essentially any other biomedical resource. Nanopublication of LWAS results are increasingly essential when confronting inherently complex biological systems where both the numbers of biomedical concepts and the resulting combinatorial explosion of possible associations lie beyond human knowledge and reasoning capacity.

## Materials and Methods

We start by briefly describing the algorithms behind concept profiles (for an extensive description see [9]), followed by the manual assessment steps for validating the top 105 associations, the filtering steps used in the GWAS study, and the method and design choices behind exposing the gene-disease associations as nanopublications. We end with an overall description of the software and pipeline.

### Concept profile generation

Exposing the network of implicit information from text was a three-step procedure involving concept indexing, concept profile creation and concept profile matching. First, we applied an Open Source concept recognition software called Peregrine employing simple contextual heuristics to resolve homonyms and map ambiguous protein, gene and disease names and spellings to unique biomedical concepts [46]. Peregrine has been shown to have F-measures (harmonic mean of precision and recall) of 0.765 for gene mentions [46] and 0.569 for disease mentions [35]. Other than the recognition of terms, we employed no other natural language processing procedures, such as noun or verb recognition. Coupled to Peregrine we used an extensive curated in-house thesaurus of biomedical concepts based on the 2010AB UMLS [47] augmented with concepts from Entrez Gene [48], OMIM, UniProt [49], the Human Gene Nomenclature Database [50], and JoChem [51]. The gene thesaurus was expanded so as to improve recall by taking into account synonyms due to common spelling variations [52]. The UMLS thesaurus was adapted for efficient natural language processing by avoiding terms that were overly ambiguous, and complicated clinical terms not likely to be found in text [53]. We also excluded UMLS semantic categories considered irrelevant for biological information about genes [10]. The thesaurus contained 687,718 biomedical concepts in total, of which 19,113 are genes from different species and 21,847 are diseases. Using Peregrine, over 17 million MEDLINE documents (titles, Medical Subject Headings, and abstract text) dating from January 1980 up to the stated time points were then indexed. Stop words were removed and words were stemmed to their uninflected form by the lexical variant generator normalizer [54].

In the second step, concept profiles were constructed for all concepts. A concept profile is an *M*-dimensional vector w_x_ = (*w*_x1_,*w*_x2_,…,*w*_*xM*_) where *x* is a particular concept and *M* is the total number of concepts in the thesaurus. The weight *w*_*xy*_ indicates the strength of the association of concept *x* to concept *y* and is computed from pairwise concept-concept co-occurrence frequencies within individual abstracts. Co-occurrence is characterized by four contingencies: both concepts occur, neither concepts occurs, or one concept occurs without the other. Based on these contingencies, association strength between *x* and *y* is computed using the symmetric uncertainty coefficient [5,55]. This information-theoretical measure takes the *a priori* probability of direct relations into account, and is a way to normalize co-occurrence statistics for literature and concept usage biases. In particular, it gives extra weight to those concepts that have very specific associations. As an example, consider the concept *DMD* (the gene) and the corresponding disease Duchenne Muscular Dystrophy. In the vast majority of MEDLINE abstracts, both concepts will be absent. However, there will still be many abstracts where these concepts appear together. Relatively few abstracts will mention one concept but not the other. This results in a high value for the uncertainty coefficient reflecting the strong association between *DMD* and Duchenne. In contrast, the concepts “human” and *DMD* will yield low association score. In this case, “human” is a generic concept and there will be many abstracts where human and *DMD* occur together, but also many other abstracts where human occurs regardless of any other concept. We know from previous [13] and related [56] work that co-occurrence within the same abstract is, with some margin of error, an effective indicator of associations made explicit by the author (consider the earlier example of the mutated *DMD* gene causing the disease Duchenne Muscular Dystrophy).

In the third and last step, we use the previously established explicit associations between concept pairs to expose implicit associations from the text. To do this we compute an association score between concepts based on the similarity or match between their concept profiles. This match score is based on the connecting concepts, i.e., those concepts that appear in the concept profiles for both X and Y (even if X and Y themselves never co-occur explicitly in literature). The match score is implemented as the inner product of two concept vectors, so a higher match score indicates a stronger association (reflecting the number of shared concepts between X and Y and the weight (*w*_*xy*_) of those concepts). We then calculate the relative association as a percentile rank over the ensemble of associations.

### Manual assessment steps

The 105 highest ranking implicit gene-disease associations and their top connecting concepts were manually assessed by searching the Online Mendelian Inheritance in Man (OMIM) (14) and PubMed databases with an expanded synonym search for information about the gene and the disease separately, in conjunction with the top connecting concept (see S4 Table).

### GWAS catalogue filtering steps

Gene-disease associations were removed if they 1) were already reported in earlier studies as a GWAS hit in the GWAS catalogue, 2) mentioned “response (to)” as part of the disease/trait description or 3) could not be mapped unambiguously to a gene or disease concept from our thesaurus using the gene symbol as reported by the authors of the study. Mapping of gene symbols and disease descriptions to thesaurus concepts was achieved by querying the Peregrine web service [http://implicitome.cloud.tilaa.nl/conceptlinker/service/index/indexText]. This service recognizes concepts in free text and uses the same indexing engine as used for generating the concept profiles. Queries to the service return matching concepts, their semantic types and a number of external database identifiers. These identifiers reference major biomedical data sources, such as GO and UMLS. Concept suggestions with the correct semantic type, i.e. “gene” (T028) or “disease” (T047), were mapped back to the corresponding concept in our thesaurus using the “mapDatabaseIDListToConceptIDs” method of the Concept Profile Analysis (CPA) web service [57]. Case sensitive matches between GWAS gene/disease description and concept label was preferred when more than one concept was suggested. For all GWAS entries that were successfully mapped to concept pairs, we removed those that had an explicit co-occurrence in literature and those that did not have a concept profile. This was determined by utilizing the “findCoOccurringDocuments” and the “filterConceptsWithProfile” CPA methods respectively. For the remaining entries, match scores were retrieved by invoking the “getSimilarConceptProfiles” method, and mapped to a percentile rank by projection onto the match scores of 1500 randomly selected gene-disease associations that were retrieved from the same service. For duplicate GWAS gene-disease associations, only the p-value of the most significant observation was considered. GWAS hits with p-values smaller than 10^−8^ were classified as “high significance”, hits with p-values between 10^−8^ and 10^−5^ as “intermediate significance”.

### Exposing LWAS as nanopublications

In order to promote reuse and machine-readability, we exposed gene-disease associations from our LWAS as nanopublications [43] following the guidelines of the Open PHACTS consortium [58] (available at http://nanopub.org/guidelines). Each of these nanopublications has an identical format describing the LWAS-derived association for a gene-disease pair. At its highest level of description, a nanopublication has three essential components: an Assertion, Provenance, and PublicationInfo. The Assertion contains statements composing a single scientific claim. The Provenance contains statements about how the Assertion “came to be” including attribution and the methodological context from which the Assertion was derived helping others to estimate the trustworthiness of the claim. PublicationInfo documents attribution to the creators (people and/or institutions) of the nanopublication itself. PublicationInfo also includes citation and reuse information such as date and time-stamp, licensing and versioning information.

At the level of implementation, a nanopublication is a schema built on top of existing semantic technology that uses statements in the form of subject-predicate-object triples to encode associative knowledge. The statements are serialized using the Resource Description Framework (RDF) such that all the entities are identified using resolvable Uniform resource identifiers (URIs). URIs ensure machine readability and data interoperability, and permit automated search and reasoning. The nanopublications herein are composed of a total of 20 triples employing URIs from seven existing public ontologies.

The Assertion in our gene-disease nanopublication is a claim that a particular gene has an association with a particular disease with a relative strength denoted by a percentile rank. The SemanticScience Integrated Ontology (SIO) provided the key ontological terms when modeling this association. The gene and the disease in the association are identified using URIs from an RDF version of the Peregrine thesaurus that was used to index MEDLINE in the first step of our concept profile pipeline. Using these thesaurus URIs it is possible to retrieve for example concept definitions, labels and identifiers under which the concept is known in its original data sources: e.g. MeSH, OMIM or ChEBI. Note that the latter feature promotes interoperability of the data contained in the nanopublications: the associations that are exposed by the nanopublications can relatively easily be integrated with any other dataset using those external identifiers.

The provenance model of the gene-disease nanopublication links to an RDF resource identifier that denotes the process from which the assertion was derived. In this case it refers to the concept profile-based method that determined the association with the specified relative strength as indicated by the percentile score. We note that more detailed provenance could be attached to the nanopublication provenance. For example, an extended provenance model could link to the source code repository and the specific code version, thus enabling a full provenance trace for the nanopublication assertion.

The whole implicitome contains 204,072,376 nanopublications, of which 202,592,481 have no co-occurrence in the literature. We provide an additional set of nanopublications representing the explicitome: gene disease pairs that Peregrine indexing identified to be in the same abstract. This set contains 7,858,449 nanopublications, each of which asserts a single gene-disease co-occurrence and the PubMed ID of the publication in which they co-occur.

The RDF for both the implicitome and explicitome nanopublication datasets is available through the data repository (doi:10.5061/dryad.gn219/5 and doi:10.5061/dryad.gn219/4). In addition, the associations are provided as a CSV file (doi:10.5061/dryad.gn219/6). Example nanopublications can be viewed using the nanopublication viewer that is available at http://rdf.biosemantics.org/ under the tab “Nanopublications”. Clicking on the URI will display the nanopublication in the viewer at the bottom of the page. Two more visualization options are available on the right side of the URI link, one using a graph viewer and another that will simply render the content in the browser.

### Software

**Fig 5:**
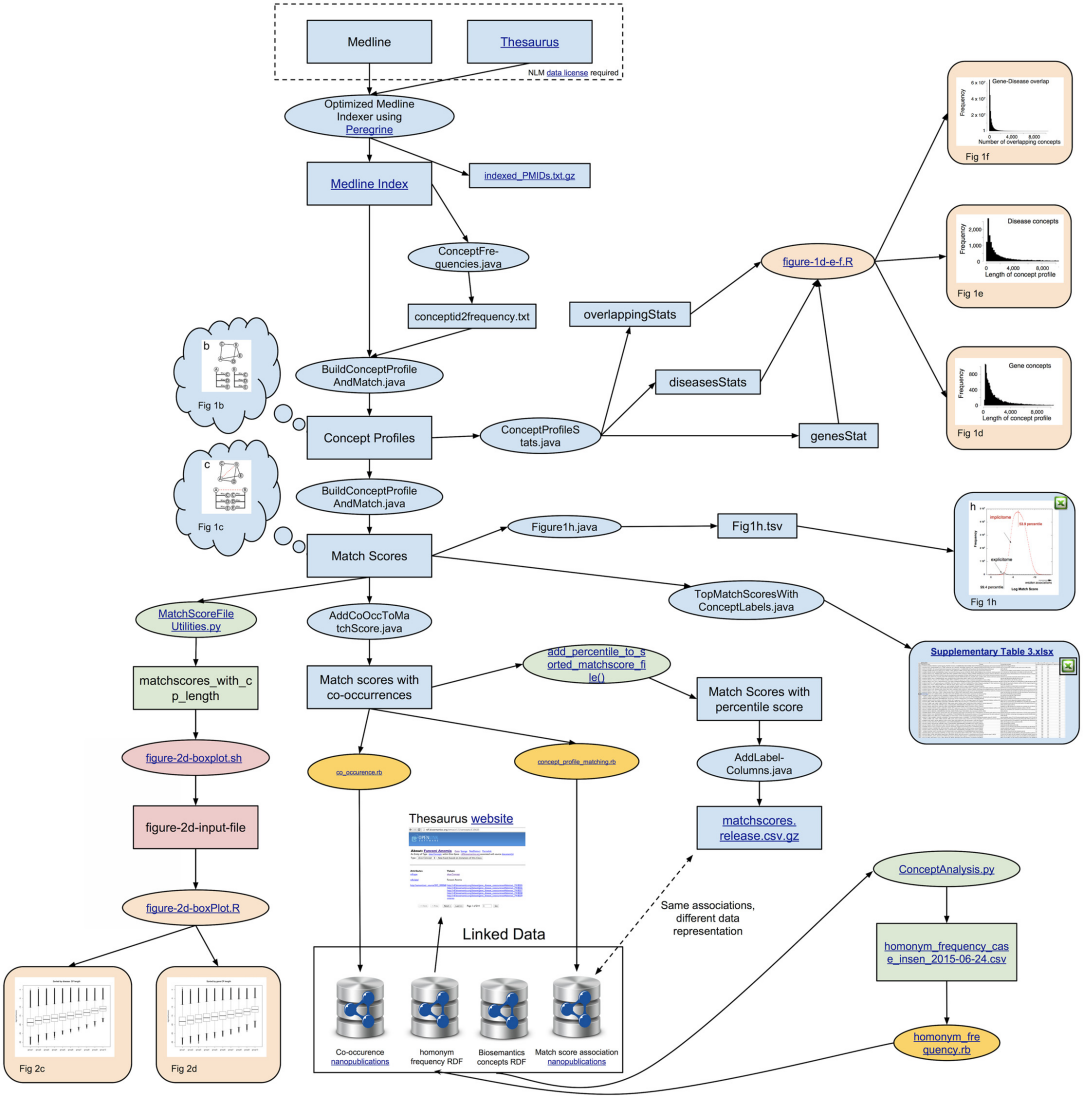
Overview of LWAS workflow (concept profile creation and analysis).

The datasets and graphs in this paper were created using a software pipeline consisting of a variety of programming languages, tools and data sources. In this section we provide a summary description to facilitate reproduction and extension of this work. Fig 5 shows an overview of the software pipeline, which starts at the top with the MEDLINE data: subsequent steps going down and sideways. A version of this figure with clickable links to respective code and data repositories is available (doi:10.5061/dryad.gn219/3). Data resources and intermediate data processing products are shown as rectangles, while the ovals show different software components (colored by programming language). Detailed documentation can be found in the source code for each component at the public Github code repository at https://github.com/BiosemanticsDotOrg/GeneDiseasePaper.

All software components and datasets are available under an open source license, with the exception of the MEDLINE corpus and the (UMLS-based) thesaurus. For these, a license is required that may be obtained from the NIH U.S. National Library of Medicine. Information about individual concepts from our thesaurus can be found at http://rdf.biosemantics.org/fct. Our code contains a modified version of the Peregrine indexer, which is available in the Github repository. The standard Peregrine version is available from https://trac.nbic.nl/data-mining. A Berkeley DB key-value store was used to achieve efficient storage and retrieval of the indexed concepts. It contains both concept ID to PMID mappings (“in which abstract does the concept occur?”) and PMID to concept ID mappings (“which concepts occur in a given abstract?”). For easier recreation of the concept profiles, match scores and subsequent analyses, we make this database available as a download (doi:10.5061/dryad.gn219/2), so that the time-consuming MEDLINE indexing step may be skipped.

## Supporting Information

S1 Table Distribution of match score values. Histogram of the complete range of match scores for both implicit and explicit associations.
(XLSX)

S2 Table Literature abundance for genes. Comma separated: concept ID, label and number of occurrences.
(CSV)

S3 Table Literature abundance for diseases. Comma separated: concept ID, label and number of occurrences.
(CSV)

S4 Table Details for 105 highest ranking implicit associations. Gene and disease by label; connecting concepts (contribution to match score percentage); classification.
(XLSX)

S5 Table GWAS records after filtering. (XLSX)

S6 Table Nanopublication-compatible resources. (XLSX)
